# Small for gestational age: Case definition & guidelines for data collection, analysis, and presentation of maternal immunisation safety data

**DOI:** 10.1016/j.vaccine.2017.01.040

**Published:** 2017-12-04

**Authors:** Elizabeth P. Schlaudecker, Flor M. Munoz, Azucena Bardají, Nansi S. Boghossian, Asma Khalil, Hatem Mousa, Mirjana Nesin, Muhammad Imran Nisar, Vitali Pool, Hans M.L. Spiegel, Milagritos D. Tapia, Sonali Kochhar, Steven Black, For The Brighton Collaboration Small for Gestational Age Working Group

**Affiliations:** a Cincinnati Children’s Hospital Medical Center, Division of Infectious Diseases and Global Health Center, Cincinnati, OH, USA; b Baylor College of Medicine, Department of Pediatrics and Molecular Virology and Microbiology, Houston, TX, USA; c ISGlobal, Barcelona Ctr. Int. Health Res. (CRESIB), Hospital Clínic – University of Barcelona, Barcelona, Spain; d Department of Epidemiology & Biostatistics, Arnold School of Public Health, University of South Carolina, Columbia, SC, USA; e Fetal Medicine Unit, St George’s Hospital, University of London, UK; f Fetal Medicine Unit, University Hospital of Leicester, UK; g Office of Clinical Research Resources, NIAID/NIH, Rockville, MD, USA; h Aga Khan University, Department of Pediatrics and Child Health, Karachi, Pakistan; i Sanofi Pasteur, Swiftwater, PA, USA; j Kelly Government Solutions, Contractor to DAIDS/NIAID/NIH, Rockville, MD, USA; k Center for Vaccine Development, University of Maryland School of Medicine, Baltimore, MD, USA; l Global Healthcare Consulting, Delhi, India; m Erasmus University Medical Center, Rotterdam, The Netherlands

**Keywords:** Small for gestational age, Adverse event, Maternal immunisation, Guidelines, Case definition, Brighton collaboration, GAIA

## 1 Preamble

### 1.1 Need for developing case definitions and guidelines for data collection, analysis, and presentation for small for gestational age (SGA) as an adverse event following maternal immunisation

Small for gestational age (SGA) fetuses or newborns are those smaller in size than normal for their gestational age, most commonly defined as a weight below the 10th percentile for the gestational age. This classification was originally developed by a 1995 World Health Organization (WHO) expert committee, and the definition is based on a birthweight-for-gestational-age measure compared to a gender-specific reference population [1], [2].

Successful pregnancy, including optimal growth of the fetus, relies on a careful balance between immune tolerance and suppression. Several mechanisms work together to protect the fetus from rejection [3]. During normal placentation, several changes occur, including differentiation of the endometrium to decidua, development of the fetal placental trophoplast to invade the decidua, migration and differentiation of trophoblast, and remodeling of the uterine arteries [4]. Current evidence suggests that the placenta creates a micro-environment that controls immune cell differentiation at the implantation site and trophoblastic cell-induced differentiation of the immune cells into a phenotype beneficial for the trophoblast [5]. Mor and Cardenas categorized pregnancy into three different immunological stages [6]. The first pro-inflammatory phase, occurring during the first trimester, includes implantation and placentation. It is associated with increased levels of interleukin (IL)-8, macrophage chemo-attractor protein 1 (MCP-1), and activated T cells. The second anti-inflammatory phase, occurring during mid-pregnancy, is a unique period of fetal growth and development. It is characterized by predominant anti-inflammatory cytokines (IL-4, IL10 and IL-13). The third pro-inflammatory phase is similar to the first phase, and it is a preparatory stage for delivery [3]. Furthermore, different Pattern Recognition Receptors (PRRs), including Toll-like receptors and Nod-like receptors, and the innate immune system play a vital role in this process.

Dysfunction of the maternal innate immune response may predispose to placentally mediated diseases such as pre-eclampsia (PET), fetal growth restriction (FGR), placental abruption, and intrauterine fetal death. The complement system can affect angiogenesis-related endothelial cell function. It can indirectly, through macrophages, upregulate the anti-angiogenic soluble vascular endothelial growth factor receptor-1 (SFlt-1). In addition, SFlt-1 can combine with soluble endoglin (sEnd) to induce PET, FGR, and coagulation defects [7], [8], [9], [10].

Traditionally, the causes for “pathological” growth restriction are subdivided into fetal, placental and maternal. Genetic and chromosomal disorders, fetal malformation, infection (e.g. rubella or cytomegalovirus), and toxic substances (e.g. alcohol, cocaine, or smoking) can contribute to FGR. Maternal diseases such as anemia and malnutrition may also affect fetal growth. However, classical utero-placental dysfunction accounts for the vast majority of cases of “placental” FGR, as well as to a variety of conditions such as pre-eclampsia and placental abruption [11]. The Brighton Collaboration fetal growth restriction manuscript addresses the impact of obstetric conditions on fetal growth restriction more fully [12].

Congenital infections by *Toxoplasma gondii*, rubella, cytomegalovirus, herpes simplex virus (HSV), varicella-zoster virus, *Treponema*, and HIV contribute to 5–10% of fetal growth restriction [13], [14]. Several investigators believe that congenital infection could be associated with a spectrum of disease, and it could be quite variable, ranging from severe clinical manifestations to mild disease only presenting with a small for gestational age fetus. Many clinicians think that TORCH screens should be performed on every SGA newborn infant [15], [16], [17].

Placental malaria is a major cause of fetal growth restriction. In a case-control study of 492 pregnant Malawian women, a significant increase of placental complement C5a levels was associated with an increased risk of delivering a small-for-gestational-age infant [18]. C5a was significantly increased in placental malaria and was negatively correlated with the angiogenic factor angiopoietin-1 and positively correlated with angiopoietin-2, soluble endoglin, and vascular endothelial growth factor [18].

Maternal vaccination during pregnancy has emerged as a recommended public health approach to prevent maternal and childhood infections. All current maternal vaccines were initially designed for and tested in non-pregnant populations, but the diverse immune modulations during pregnancy may cause pregnant women to respond sub-optimally or differently compared with non-pregnant populations [19]. In addition, vaccine efficacy could be affected by other factors including the dose, route, and timing of the vaccination. Limited data exist on the effect of vaccinations in high-risk pregnancies. In spite of the success of several maternal vaccines, many gaps exist in our knowledge of this promising public health strategy and impact on fetal growth during pregnancy.

Tetanus and influenza vaccines were the first vaccines recommended for use during pregnancy. Trotta and colleagues evaluated the safety of A/H1N1 pandemic vaccination of 6246 pregnant women [20]. There was no difference in pregnancy outcome measures, including small for date. In an observational cohort study from UK, Donegan and colleagues examined maternal and neonatal outcomes among 6185 pertussis vaccinated pregnant women and 18,523 healthy unvaccinated historic controls [21]. There were no significant differences between the two groups regarding low birth weight or other maternal and neonatal outcomes [21], and these findings were confirmed by others [22]. Currently, the World Health Organization (WHO) provides guidance for vaccination during pregnancy (Table 1). The key question that remains is related to the safety and optimum timing of vaccination and if maternal vaccination has any negative effects on the immune system [23].

**Table 1:** Summary of vaccines reviewed and level of evidence concerning vaccine safety.

Vaccine	Increased risk or severity of disease in pregnant women	Risk of disease to fetus or young infant	WHO recommendation on vaccination during pregnancy	Vaccine safety concerns	Level of evidence on vaccine safety
*Inactivated vaccines*					
Seasonal TIV or H1N1 2009–2010 monovalent, nonadjuvanted vaccines	More severe disease especially in second and third trimester and increased risk of death in a pandemic	Possible increased spontaneous abortion rate and increased preterm delivery. No malformations confirmed	Yes	No SGA safety concerns identified	++++
Oil-in-water adjuvanted, monovalent H1N1 vaccines			Yes	No SGA safety concerns identified	+++
Tetanus toxoid vaccines	Incidence depends on region; unaltered by pregnancy	Neonatal tetanus mortality 60%	Yes	No SGA safety concerns identified	++
Meningococcal polysaccharide vaccines	Incidence not altered by pregnancy	Unknown for fetus; infants may develop significant morbidity and mortality.	No	No SGA safety concerns identified	++
Meningococcal conjugate vaccines			As part of mass campaigns.	No SGA safety concerns identified	+

*Live attenuated vaccines*					
Rubella vaccine	Incidence not altered by pregnancy	Abortion and congenital rubella syndrome (CRS)	No	No SGA safety concerns identified	+++
Measles vaccines	More severe disease; low mortality	Possible higher abortion rate, infrequently congenital measles and if premature possible high case fatality rate	No	No SGA safety concerns identified	Indirect data from combined MR vaccines
Mumps vaccine	Incidence not altered by pregnancy	Probable increased rate of abortion in the first trimester	No	No SGA safety concerns identified	Indirect data from combined MR vaccines
Oral poliovirus vaccine	Increased risk of paralytic disease	Anoxic fetal damage reported; 50% mortality in neonatal disease	No	No SGA safety concerns identified	+++
Yellow fever	Incidence not altered by pregnancy	Unknown	During epidemics and when travel to endemic areas cannot be avoided	No SGA safety concerns identified	+++

+++ Evidence from observational studies or registries with pregnancy follow-up and passive surveillance.

++ Some evidence from studies with lower power, lack of information on some relevant pregnancy outcomes, short follow-up of offspring or other limitations of study design and passive surveillance.

+ Passive surveillance data.

– No data.

Placentally mediated severe FGR, indicated by abnormal uterine and umbilical artery Doppler velocimetry, is associated with impaired transplacental gas transfer and fetal hypoxaemia. This triggers compensatory re-distribution of blood towards essential organs (brain, heart, and adrenals) and decreases blood flow to other organs (kidneys and bowel). This “compensatory phase” can be recognised by observing Doppler changes (reduced resistance) in the middle cerebral artery (MCA), decreased amniotic fluid, and/or bright echogenic bowel. The duration of this compensatory phase is variable. This phase is followed by a phase of myocardial dysfunction and haemodynamic decompensation. This “decompensation phase” can be recognised by abnormal venous Doppler waveforms (absent or negative ‘a’ wave) and it is associated with fetal acidaemia. Both hypoxaemia and acidaemia can also be detected clinically by changes in fetal heart rate as well as the biophysical profile. The Brighton Collaboration growth restriction and fetal distress guidelines further explore these issues [12], [24].

Despite the presence of many pathophysiological events that may lead to intrauterine growth restriction, SGA is not universally associated with growth restriction. Small for gestational age (SGA), is commonly used as a proxy for intrauterine growth restriction (IUGR), particularly in settings where serial ultrasonography is not readily available [2], [25]. However, fetuses that are SGA are not necessarily growth restricted; they in fact may be constitutionally small. If SGA babies have been the subject of intrauterine growth retardation (IUGR), the term “SGA associated with IUGR” is used. IUGR refers to a fetus that is unable to achieve its genetically determined potential size. This functional definition aims to identify a population of fetuses at risk for poor pregnancy outcomes, and excludes fetuses that are SGA but are not pathologically small. Neonates born with severe SGA (or with severe short stature) are defined as having a length less than 2.5 standard deviation below the mean [26].

A related term is low birth weight (LBW), defined as a birth weight of less than 2500 g, regardless of gestational age at the time of birth. Additional related terms include very low birth weight (VLBW) which refers to less than 1500 g, and extremely low birth weight (ELBW) which is less than 1000 g. Normal weight at term delivery is 2500–4200 g. LBW is discussed further in a separate document for this definition. It is important to be clear that SGA is not a synonym of LBW, VLBW or ELBW. Approximately one third of LBW babies weighing less than 2500 g are also SGA [12], [27].

In this case definition and associated guideline, we propose a systemic tool for evaluating the adverse event of SGA after maternal immunisation. It is important to emphasize that these tools have been developed in the absence of any data supporting such an association but rather to facilitate studies of the safety of vaccines used in pregnancy. The outcome of SGA has been examined in several published studies of the safety of influenza and pertussis vaccination during pregnancy. In one randomised clinical trial of influenza immunisation in pregnant women in Bangladesh, SGA was defined as less than 10th percentile weight for gestational age [28], [29]. In this trial, two reference standards were used - the reference values for distributions of birth weights from the United States [30] and the global reference standard from the World Health Organization [31].

The remaining published studies were observational in design and used different SGA definitions and reference standards (Table 2) [32], [33]. In most of these studies, SGA was defined as the lowest 10th percentile of the gestational age-specific birth weight within the cohort of live births, not stratified or stratified by gender [20], [22], [34], [35], [36], [37], [38], [39], [40], [41], [42], [43], [44], [45], [46], [47], [48]. In two studies, in addition to the 10th percentile cut-off, the investigators used other cut-offs to define very small neonates, like low 3rd and low 5th percentiles [49], [50]. In three studies, SGA was defined as a births weight below 2 standard deviations from the national reference mean [51], [52], [53].

**Table 2:** Selected publications discussing maternal and fetal outcomes of vaccination during pregnancy and mentioning the outcome of Small for Gestational Age (SGA), among articles published between 1 January 2006 and 1 June 2016. PubMed search strategy: (pregnancy OR pregnant) AND (vaccine OR vaccination OR immunisation) AND (pertussis OR Tdap OR tetanus OR influenza); N = 1402 articles retrieved; 26 relevant publications extracted (by Vitali Pool, MD).

Reference	SGA definition	Vaccine	#
Schatz et al. (2011)	SGA defined as birth weight for gestational age <10th percentile	Any	[48]
Adedinsewo et al. (2013)	SGA defined as the lowest 10th percentile of birth weight for each gestational week stratified by infant sex (reference values from US data^a^	IIV	[34]
Ahrens et al. (2014)	<10th percentile in weight for sex-specific gestational age	IIV	[35]
Baum et al. (2015)	SGA; birth weight and/or length more than 2 SD below the sex- and gestational age-specific reference mean^b^	IIV	[51]
Beau et al. (2014)	SGA was defined as any singleton with a birth weight <2 standard deviations (SD) from the French reference weight mean, adjusted for gestational age and sex^c^	IIV	[52]
Cantu et al. (2013)	Defined by Brenner’s standard for fetal growth <10th percentile^d^	IIV	[36]
Chambers et al. (2013)	<10th centile for sex and gestational age in live born infants using standard U.S. growth charts for full and preterm infants^e^	IIV	[37]
Dodds et al. (2012)	⩽10th percentile	IIV	[38]
Fell et al. (2012)	Two definitions were used to report fetal outcomes: (1) Small for gestational age: below 10th percentile, and (2) Small for gestational age: below 3rd percentile	IIV	[49]
Huang et al. (2014)	SGA defined as live birth with birth weight <10th percentile for their gestational age (using Taiwan reference values for fetal birth weights)^f^	IIV	[39]
Källén et al. (2012)	SGA defined as <2 SD from expected weight at the relevant gestational week.	IIV	[53]
Legge et al. (2014)	SGA defined as the bottom 10th percentile of birth weight [for each sex] for each week of gestational age	IIV	[41]
Ludvigsson et al. (2013)	SGA defined as <10th percentile of the gestational age-specific birth weight within the cohort	IIV	[42]
Nordin et al. (2014)	Authors defined 2 cutoffs for SGA birth, <10th and <5th percentiles. Reference values for distributions of birth weights were derived from US data, stratified by sex^a^	IIV	[50]
Olsen et al. (2016)	Gestational age was calculated by last menstrual period captured at the time of the first antenatal care visit. SGA was calculated using the Kramer method, defined as a live birth with a birth weight <the 10th percentile of birth weights of the same sex and same gestational age in weeks^g^	IIV	[32]
Omer et al. (2011)	Below the 10th percentile	IIV	[43]
Pasternak et al. (2012)	Lowest 10th percentile of the gestational age-specific birth weight within the cohort	IIV	[44]
Richards et al. (2013)	SGA not defined, but likely was <10th percentile weight for gestational age as used by the same authors in other studies/publications	IIV	[45]
Trotta et al. (2014)	Live newborns with birth weight below the 10th centile for their gestational age within the cohort of live births only	IIV	[20]
van der Maas et al. (2016)	SGA defined as a birth weight below the tenth centile, adjusted for gestational age and based on Dutch averages^i^	IIV	[47]
Steinhoff et al. (2012)	<10th percentile weight for gestational age; two reference standards were used – the reference values for distributions of birth weights from US^a^ and the Global reference standard by WHO^h^	IIV vs PPSV	[28]
Steinhoff et al. (2012)	<10th percentile weight for gestational age	IIV vs PPSV	[29]
Berenson et al. (2016)	Below the 10th percentile	Tdap	[22]
Kharbanda et al. (2014)	<10th percentile weight for gestational age	Tdap	[40]
Morgan et al. (2015)	SGA outcome is reported but not clearly defined	Tdap	[33]
Sukumaran et al. (2015)	Less than the 10th percentile for gestational age and sex	Tdap	[46]

NOTE: IIV – Inactivated Influenza Vaccine; Tdap – diphtheria, tetanus and acellular pertussis vaccine; PPSV – Pneumococcal Polysaccharide Vaccine.

a Oken E, Kleinman KP, Rich-Edwards J, Gillman MW. A nearly continuous measure of birth weight for gestational age using a United States national reference. BMC Pediatr. 2003 Jul 8;3:6. Epub 2003 Jul 8. (http://www.biomedcentral.com/1471-2431/3/6http://www.biomedcentral.com/1471-2431/3/6 accessed 31 May 2016)

b Sankilampi U, Hannila ML, Saari A, Gissler M, Dunkel L. New population-based references for birth weight, length, and head circumference in singletons and twins from 23 to 43 gestation weeks. Ann Med 2013;45(5–6 (Sep)):446–54.

c Salomon L-J, Bernard J-P, de Stavola B, Kenward M, Ville Y. Poids et taille denaissance: courbes et équations. J Gynecol Obstet Biol Reprod 2007;36(1):50–6.

d Brenner WE, Edelman DA, Hendricks CH. A standard of fetal growth for the United States of America. Am J Obstet Gynecol 1976;126:555–64.

e Lubchenco LO, Hansmann CO, Dressler M, Boyd E. Intrauterine growth as esti-mated from liveborn birthweight data at 24 to 42 weeks of gestaton. Pediatrics1963;32:793–800. *Also in* Lubchenco LO, Hansmann C, Boyd E. Intrauterine growth in length and headcircumference as estimated from libirth at gestational ages from 26 to 42 weeks. Pediatrics 1966;37:403–7.

f Hsieh WH, Wu HC, Jeng SF, et al. Nationwide singleton birth weight per-centiles by gestational age in Taiwan, 1998–2002. Acta Pediatr Taiwanica2006;47:25–33.

g Kramer MS, Platt RW, Wen SW, Joseph KS, Allen A, Abrahamowicz M, Blondel B, Bréart G; Fetal/Infant Health Study Group of the Canadian Perinatal Surveillance System. A new and improved population-based Canadian reference for birth weight for gestational age. Pediatrics. 2001 Aug;108(2):E35.

h WHO Multicentre Growth Reference Study Group. WHO child growth standards: methods and development. Geneva (Switzerland): World Health Organization; 2006. (www.who.int/childgrowth/standards/technical_report/en/www.who.int/childgrowth/standards/technical_report/en/ accessed 2011 Nov. 15).

i Visser GH, Eilers PH, Elferink-Stinkens PM, Merkus HM, Wit JM. New Dutch reference curves for birthweight by gestational age. Early Hum Dev. 2009 Dec;85(12):737–44.

Among the national population-based weight references used by the investigators, two older US population based standards were mentioned [54], [55], as well as population references for Taiwan [56], France [57], Canada [58], The Netherlands [59], and Sweden [53]. No published studies, however, provided details of how the birth weight was measured (e.g. the type of scales used) or specified the age (in days) at which the measurements were taken.

The WHO definition of SGA, outlined by a 1995 WHO expert committee, remains the most widely utilized definition of SGA [1], [2]. This classifies SGA infants as having a birth weight for gestational age below the 10th percentile based on a sex-specific reference population. Some infants classified as SGA will include newborns who are constitutionally small and are in the lower tail of the growth curve distribution, while others will include newborns who are growth restricted in utero due to one or more growth-inhibiting factors such as malnutrition, placental insufficiency, pregnancy complications as preeclampsia, and/or infection [2], [60]. Other less utilized SGA definitions in the literature include having a birth weight 2 standard deviations below the standard for gestational age or a birth weight for gestational age below the 5th or 3rd centile [60], [61]. Using a more restrictive than a liberal definition captures more severe cases of SGA rather than infants who are constitutionally small.

Essential to the SGA definition is accurate dating of gestational age [62], [63] and accurate assessment of birth weight [27], [61]. Early ultrasound (accuracy ±5 days if first trimester and ±7 days after first trimester), ideally in the first trimester, is the gold standard for gestational age assessment [62], [64]. Gestational age assessment based on last menstrual period (LMP) date has lower accuracy (±14 days) given different cycle duration in women, ovulation/conception timing, and recall error [65]. The accuracy of newborn physical examination (±13 days for Dubowitz) is influenced by the complexity of the score used and the examiner’s expertise level [64].

A main challenge in defining SGA is selecting the appropriate comparison population [2], [60], [66]. It is important to select the appropriate comparison charts as the rates of SGA can differ significantly based on the choice of the reference population [66], [67]. There are two types of charts available including reference and standard charts. Standard charts are prescriptive and delineate how a population should grow under optimal environmental and health conditions and are based on low-risk pregnancies [68], [69]. Reference charts are descriptive, include both low-risk and high-risk pregnancies, and specify growth in a particular place and time [68], [69]. A recent standard sex-specific birth weight for gestational age chart using neonatal growth measures from healthy women in eight countries with ultrasound assessed gestational age has been published by the INTERGROWTH-21st Project group [70]. This standard, based on a low-medium risk group of pregnant women, is for infants born at 33–42 weeks’ gestation as too few women gave birth to infants prior to 33 weeks [71]. The majority of the charts in the literature are reference charts that differ considerably in terms of sample size, population characteristics, representativeness (hospital based versus population-based), inclusion/exclusion criteria, and methods of gestational age assessment [2], [66]. A recent meta-analysis identified 26 commonly cited reference charts which include the 10th percentile cut point to define SGA status [66]. The majority of the available reference populations were from North America (n = 12) with some from Europe (n = 6) and Asia (n = 5), and few from Africa (n = 2) and South America (n = 1) [66]. The majority of the charts used LMP dates to define gestational age reported to the nearest week, while some used ultrasound or best obstetric estimate [66].

Customizing fetal size for maternal height, weight and ethnicity, has been shown to improve the identification of babies who are small because of FGR, rather than constitutional reasons [72], [73], [74], [75], [76], [77]. However, although maternal height, weight and ethnicity are significant predictors of fetal size, they do not explain a large proportion of the birth weight variability [78], [79], and therefore, their utility for customization is limited. Furthermore, it is important to point out a major criticism to the customized fetal growth charts. Arguably, their reported improved ability to identify fetuses at risk of adverse outcome appears to be a consequence of an artifact rather than a real improvement in predictive ability [78], [80], [81], [82], [83], [84]. The use of Hadlock’s proportionality formula to construct these customized charts results in a substantially higher proportion of preterm infants being identified as SGA. When this artifactual identification of preterm infants is taken into account, the reported benefits of customization disappear. It is likely though that the use of customized charts is associated with improved identification of SGA fetuses of mothers who smoke, have a high body mass index or other pathologies [85].

Additionally important for preterm infants, the birth weight for gestational age reference charts differ from ultrasound-based fetal weight charts. Preterm infants are known to be smaller than in utero infants given the underlying pathological determinants, such as preeclampsia and other hypertensive disorders that impair fetal growth and increase the risk of preterm birth [86], [87], [88]. As such, the birth weight for gestational charts underestimate the prevalence of IUGR [89].

Although there is a broadly accepted definition of SGA, the aforementioned complexities involved in its evaluation, including the accurate measurement of birth weight, the determination of gestational age, and the reference chart used to analyze these data, indicate that a standardized definition of SGA is desirable. This need is especially apparent in studies of maternal immunisation where SGA may be a critical endpoint, either as a positive outcome related to vaccination or as related to safety. A positive effect of immunisation in pregnancy might be that the infant is less likely to be SGA. On the other hand, if the study vaccine somehow adversely affects maternal health or other factors that impact birth weight, then the increased incidence of SGA may indicate a safety signal. A standard definition and evaluation of SGA would support the comparability of conclusions related to the benefits and safety concerns attributed to maternal immunisation with a given vaccine across studies. Herein, to facilitate data interpretation and promote scientific understanding of the same, we propose the tools necessary for determination of SGA in the setting of clinical trials as well as post-licensure surveillance systems.

### 1.2 Methods for the development of the case definition and guidelines for data collection, analysis, and presentation for SGA as an adverse event following maternal immunisation

Following the process described in the overview paper [90], as well as on the Brighton Collaboration Website, http://www.brightoncollaboration.org/internet/en/index/process.html, the Brighton Collaboration *Small for Gestational Age Working Group* was formed in 2016 and included members of clinical, academic, public health, and industry backgrounds. The composition of the working and reference group as well as results of the web-based survey completed by the reference group with subsequent discussions in the working group can be viewed at: http://www.brightoncollaboration.org/internet/en/index/working_groups.html.

To guide the decision-making for the case definition and guidelines, a literature search in PubMed was performed and identified over twenty vaccine studies in which SGA was mentioned and defined. (The search strategy used was as follows: (pregnancy OR pregnant) AND (vaccine OR vaccination OR immunisation) AND (pertussis OR Tdap OR tetanus OR influenza); article publication dates between 1 January 2006 and 1 June 2016.) All abstracts were screened for possible reports of small for gestational age following immunisation. This review resulted in a summary of 26 articles, including information on the diagnostic criteria or case definition put forth and the vaccine used (summarized in Table 2). Multiple general medical, paediatric and infectious disease text books were also searched. Most publications defined SGA as the lowest 10th percentile of the gestational age-specific birth weight within the cohort of live births, not stratified or stratified by gender [20], [22], [34], [35], [36], [37], [38], [39], [40], [41], [42], [43], [44], [45], [46], [47].

### 1.3 Rationale for selected decisions about the case definition of SGA as an adverse event following maternal immunisation

#### 1.3.1 The term small for gestational age

Small for gestational age (SGA) is commonly used as a surrogate marker of low fetal growth trajectories, and is indeed associated with increased perinatal mortality and morbidity. In order to define perinatal outcomes and identify safety issues during maternal immunisation trials, this Brighton Collaboration case definition of small for gestational age focuses on recommendations to simultaneously define both birthweight and gestational age. Within the definition context, however, the three diagnostic levels must not be misunderstood as reflecting different grades of clinical severity. They instead reflect diagnostic certainty (see below).

#### 1.3.2 Related terms - small for gestational age

The working group considered several other related outcomes in its discussions. Principle consideration was given to both the assessment of gestational age and assessment of size/weight as both these formed the foundation for our definition. Accordingly, we worked closely with the GAIA workgroups assigned to assessment of prematurity [62], [63] and also for low birth weight [27]. Our definition and Brighton level classification scheme draw upon their work.

In addition, SGA, which assesses the size of the infant in relation to its maturity after birth, is closely related to inter-uterine growth retardation which assesses the growth of the fetus up until the time of birth and when present predicts for an SGA infant. These two entities, while closely related, use very different assessment tools, so our definition presented here acknowledges the work of the GAIA IUGR group [12] but did not draw upon their methods.

#### 1.3.3 Formulating a case definition that reflects diagnostic certainty: weighing specificity versus sensitivity

It needs to be re-emphasised that the grading of definition levels is entirely about diagnostic certainty, not clinical severity of an event. Thus, a clinically very severe event may appropriately be classified as Level Two or Three rather than Level One if it could reasonably be of non-small for gestational age aetiology. Detailed information about the severity of the event should additionally always be recorded, as specified by the data collection guidelines.

The number of symptoms and/or signs that will be documented for each case may vary considerably. The case definition has been formulated such that the Level 1 definition is highly specific for the condition. As maximum specificity normally implies a loss of sensitivity, two additional diagnostic levels have been included in the definition, offering a stepwise increase of sensitivity from Level One down to Level Three, while retaining an acceptable level of specificity at all levels. In this way it is hoped that all possible cases of SGA can be captured.

#### 1.3.4 Rationale for individual criteria or decision made related to the case definition

Our approach is to provide adequate diagnostic specificity without being overly restrictive with the objective of making available a definition that is applicable in high, middle, and low-income countries. Laboratory and pathology findings are not included in the case definition as these examinations are not required to meet the case definition of SGA. Assessment through imaging (ultrasound) at different gestational ages in pregnancy has been included to meet case definition for levels of certainty 1A to 3A, as ultrasound represents the most accurate method for assessment of gestational age.

#### 1.3.5 Influence of treatment on fulfilment of case definition

The Working Group decided against using “treatment” or “treatment response” towards fulfillment of the SGA case definition, as specific SGA treatments are not currently used or accepted.

#### 1.3.6 Timing post maternal immunisation

The diagnosis of SGA is made at birth after the measurement of birth weight, calculation of gestational age and ascertainment of weight for gestational age percentile when compared to a reference standard. Once the diagnosis has been made, no further change would be anticipated. It is expected that the birth weight and gestational age would have been obtained by a method consistent with the Brighton Collaboration standards.

The timing of the vaccine receipt or the interval from maternal immunisation and the neonatal birth are not elements of the SGA definition. In fact, the definition is meant to stand alone independent of whether the mother was vaccinated. A definition designed to be a suitable tool for describing relationships requires ascertainment of the outcome (e.g. SGA) independent from the exposure (e.g. vaccination). Therefore, to avoid selection bias, a restrictive time interval from vaccination to birth should not be an integral part of such a definition. Instead, where feasible, information on the timing of vaccine administration during pregnancy should be collected, assessed and reported as described in the data collection guidelines and the ascertainment of whether or not the infant is SGA and the associated Brighton level should be determined by personnel blinded to the vaccine status of the mother.

Further, SGA often occurs outside the controlled setting of a clinical trial or hospital. In some settings it may be impossible to obtain a clear timeline of the event, particularly in less developed or rural settings. In order to avoid selecting against such cases, the Brighton Collaboration case definition avoids setting arbitrary time frames.

#### 1.3.7 Differentiation from other (similar/associated) disorders

As described above, other similar disorders to SGA include IUGR. While IUGR refers to reduced growth velocity in the fetus as supported by at least two intrauterine growth assessments, SGA does not reflect fetal growth but size of the infant at birth [60], [61], [91]. Additionally, IUGR implies the presence of a pathological condition that occurs in utero and results in diminished fetal growth. An infant who is SGA however, does not imply that the infant has suffered from IUGR, and infants who experience a short duration of IUGR will not necessarily be SGA at birth [60], [61], [91].

### 1.4 Guidelines for data collection, analysis and presentation

As mentioned in the overview paper, the case definition is accompanied by guidelines which are structured according to the steps of conducting a clinical trial, i.e. data collection, analysis and presentation. Neither case definition nor guidelines are intended to guide or establish criteria for management of ill infants, children, or adults. Both were developed to improve data comparability.

### 1.5 Periodic review

Similar to all Brighton Collaboration case definitions and guidelines, review of the definition with its guidelines is planned on a regular basis (i.e. every three to five years) or more often if needed.

## 2 Case definition of small for gestational age^3^

SGA (small for gestational age) definition: weight below 10th percentile for gestational age as assessed against a validated global, regional or local standard.

### 2.1 Brighton Level 1 of diagnostic certainty

• Weight below 10th percentile for gestational age
AND
• The following used in assessment of weight:
  o Newborn weighed within 24 h of birth
  o Weight assessed using a calibrated electronic scale with 10 g resolution
AND
• The following for assessment of gestational age:
  o Certain LMP or IUI or embryo transfer date AND confirmatory ultrasound in first trimester
OR
• The following for assessment of gestational age:
  o First trimester ultrasound

### 2.2 Brighton Level 2A of diagnostic certainty

• Weight below 10th percentile for gestational age
AND
• The following used in assessment of weight
  o Newborn weighed within 24 h of birth on any scale with a <50 g resolution, tared to zero and calibrated
AND
• The following for assessment of gestational age:
  o Certain LMP with first or second trimester ultrasound
OR
  o Certain LMP with first trimester physical exam^4^

### 2.3 Brighton Level 2B of diagnostic certainty

• Weight below 10th percentile for gestational age
AND
• The following used in assessment of weight
  o Newborn weighed within 24 h of birth on any scale with a <50 g resolution, tared to zero and calibrated
AND
• The following assessment of gestational age
  o Uncertain LMP with second trimester ultrasound

### 2.4 Brighton Level 3A of diagnostic certainty

• Weight below 10th percentile for gestational age
AND
• The following used in assessment of weight
  o Infant weighed within the first 48 h of life
  o Newborn weighed on any scale with a <50 g resolution, tared to zero and calibrated
  AND
• The following assessment of gestational age
  o Certain LMP with third trimester ultrasound
  OR
  o Certain LMP with confirmatory 2nd trimester fundal height
  OR
  o Certain LMP with birthweight
  OR
  o Uncertain LMP with first trimester physical exam

### 2.5 Brighton Level 3B of diagnostic certainty

• Weight below 10th percentile for gestational age
AND
• The following used in assessment of weight
  o Infant weighed within the first 48 h of life
  o Newborn weight assessed by measuring the difference between an adult holding the infant and the adult being weighed alone on any scale
AND
• The following assessment of gestational age
  o Uncertain LMP with fundal height
OR
  o Uncertain LMP with newborn physical assessment
OR
  o Uncertain LMP with birthweight

### 2.6 Brighton Level 4 of diagnostic certainty

– Baby noted to be small, but no actual weight
– Baby with GA assessed only by infant examination
– Diagnosis extracted from billing codes or chart, with no documentation of actual birth weight or GA

### 2.7 Brighton Level 5 of diagnostic certainty

– No evidence of SGA or a confirmed diagnosis other than SGA.

## 3 Guidelines for data collection, analysis and presentation of small for gestational age

It was the consensus of the Brighton Collaboration *Small for Gestational Age Working Group* for small for gestational age (SGA) to recommend the following guidelines to enable meaningful and standardized collection, analysis, and presentation of information about SGA. However, implementation of all guidelines might not be possible in all settings. The availability of information may vary depending upon resources, geographical region, and whether the source of information is a prospective clinical trial, a post-marketing surveillance or epidemiological study, or an individual report of SGA. Also, as explained in more detail in the overview paper in this volume, these guidelines have been developed by this working group for guidance only, and are not to be considered a mandatory requirement for data collection, analysis, or presentation.

### 3.1 Data collection

These guidelines represent a desirable standard for the collection of data on availability following immunisation to allow for comparability of data, and are recommended as an addition to data collected for the specific study question and setting. The guidelines are not intended to guide the primary reporting of SGA to a surveillance system or study monitor. Investigators developing a data collection tool based on these data collection guidelines also need to refer to the criteria in the case definition, which are not repeated in these guidelines.

Guideline numbers below have been developed to address data elements for the collection of adverse event information as specified in general drug safety guidelines by the International Conference on Harmonization of Technical Requirements for Registration of Pharmaceuticals for Human Use [92], and the form for reporting of drug adverse events by the Council for International Organizations of Medical Sciences [93]. These data elements include an identifiable reporter and patient, one or more prior immunisations, and a detailed description of the adverse event, in this case, of SGA following immunisation. The additional guidelines have been developed as guidance for the collection of additional information to allow for a more comprehensive understanding of SGA following immunisation.

#### 3.1.1 Source of information/reporter

For all cases and/or all study participants, as appropriate, the following information should be recorded:

(1) Date of report.
(2) Name and contact information of person reporting^5^ and/or diagnosing the SGA as specified by country-specific data protection law.
(3) Name and contact information of the investigator responsible for the subject, as applicable.
(4) Relation to the patient (e.g., immuniser [clinician,nurse], family member [indicaterelationship], other).

#### 3.1.2 Vaccinee/control

##### 3.1.2.1 Demographics

For all cases and/or all study participants, as appropriate, the following information should be recorded:

(5) Case/study participant identifiers (e.g. first name initial followed by last name initial) or code (or in accordance with country-specific data protection laws). Full name should be used if privacy rules permit to avoid a misclassification.
(6) Date of birth, age, and sex.
(7) For infants: Gestational age and birth weight.

##### 3.1.2.2 Clinical and immunisation history

For all cases and/or all study participants, as appropriate, the following information should be recorded:

(8) Past medical history, including hospitalisations, underlying diseases/disorders, pre-immunisation signs and symptoms including identification of indicators for, or the absence of, a history of allergy to vaccines, vaccine components or medications; food allergy; allergic rhinitis; eczema; asthma.
(9) Any medication history (other than treatment for the event described) prior to, during, and after immunisation including prescription and non-prescription medication, as well as medication or treatment with long half-life or long term effect. (e.g. immunoglobulins, blood transfusion and immunosuppressants).
(10) Immunisation history (i.e. previous immunisations and any adverse event following immunisation (AEFI)), in particular occurrence of SGA after a previous immunisation.

#### 3.1.3 Details of the immunisation

For all cases and/or all study participants, as appropriate, the following information should be recorded:

(11) Date and time of immunisation(s).
(12) Description of vaccine(s) (name of vaccine, manufacturer, lot number, dose (e.g. 0.25 mL, 0.5 mL, etc.) and number of dose if part of a series of immunisations against the same disease).
(13) The anatomical sites (including left or right side) of all immunisations (e.g. vaccine A in proximal left lateral thigh, vaccine B in left deltoid).
(14) Route and method of administration (e.g. intramuscular, intradermal, subcutaneous, and needle-free (including type and size), other injection devices).
(15) Needle length and gauge.

#### 3.1.4 The adverse event

(16) For all cases at any level of diagnostic certainty and for reported events with insufficient evidence, the criteria fulfilled to meet the case definition should be recorded.

Specifically document:

(17) Clinical description of signs and symptoms of SGA, and if there was medical confirmation of the event (i.e. patient seen by physician).
(18) Date/time of onset,^6^ first observation^7^ and diagnosis,^8^ end of episode^9^ and final outcome.^10^
(19) Concurrent signs, symptoms, and diseases.
(20) Measurement/testing
  • Values and units of routinely measured parameters (e.g. temperature, blood pressure) – in particular those indicating the severity of the event;
  • Method of measurement (e.g. type of thermometer, oral or other route, duration of measurement, etc.);
  • Results of laboratory examinations, surgical and/or pathological findings and diagnoses if present.
(21) Treatment given for SGA.
(22) Outcome^9^ at last observation.
(23) Objective clinical evidence supporting classification of the event as “serious”.^11^
(24) Exposures other than the immunisation 24 h before and after immunisation (e.g. food, environmental) considered potentially relevant to the reported event.

#### 3.1.5 Miscellaneous/general

(25) The duration of surveillance for SGA should be predefined based on the relatively narrow window of time for diagnosis at birth.
(26) The duration of follow-up reported during the surveillance period should be predefined likewise. It should aim to continue to resolution of the event.
(27) Methods of data collection should be consistent within and between study groups, if applicable.
(28) Follow-up of cases should attempt to verify and complete the information collected as outlined in data collection guidelines 1 to 24.
(29) Investigators of patients with SGA should provide guidance to reporters to optimise the quality and completeness of information provided.
(30) Reports of SGA should be collected throughout the study period regardless of the time elapsed between immunisation and the adverse event. If this is not feasible due to the study design, the study periods during which safety data are being collected should be clearly defined.

### 3.2 Data analysis

The following guidelines represent a desirable standard for analysis of data on SGA to allow for comparability of data, and are recommended as an addition to data analysed for the specific study question and setting.

(31) Reported events should be classified in one of the following five categories including the three levels of diagnostic certainty. Events that meet the case definition should be classified according to the levels of diagnostic certainty as specified in the case definition. Events that do not meet the case definition should be classified in the additional categories for analysis.

#### 3.2.1 Event classification in 5 categories^12^

##### 3.2.1.1 Event meets case definition

Level 1: Criteria as specified in the SGA case definition.
Level 2: Criteria as specified in the SGA case definition
Level 3: Criteria as specified in the SGA case definition

#### 3.2.2 Event does not meet case definition

##### 3.2.2.1 Additional categories for analysis

(4) Reported SGA with insufficient evidence to meet the case definition^13^
(5) Not a case of SGA^14^
(32) The interval between immunisation and reported SGA could be defined as the date/time of immunisation to the date/time of diagnosis. It should be noted that the diagnosis of SGA is made at birth whereas any damage due to immunisation or another exposure happened during the pregnancy. Thus the interval between immunisation date and the date of diagnosis is less useful than in classical vaccine association studies.

### 3.3 Subjects with small for gestational age by interval to presentation

#### 3.3.1 Interval (diagnosis of SGA made at delivery) number/percent

Immunisation >12 weeks prior to SGA diagnosis.
Immunisation >8 and <12 weeks prior to SGA diagnosis.
Immunisation >4 and <8 weeks prior to SGA diagnosis.
Immunisation <4 weeks prior to SGA diagnosis.
Weekly increments thereafter.

## 4 Total

(33) The duration of a possible SGA could be analysed as the interval between the date/time of diagnosis or birth. Whatever start and ending are used, they should be used consistently within and across study groups.
(34) If more than one measurement of a particular criterion is taken and recorded, the value corresponding to the greatest magnitude of the adverse experience could be used as the basis for analysis. Analysis may also include other characteristics like qualitative patterns of criteria defining the event.
(35) The distribution of data (as numerator and denominator data) could be analysed in predefined increments (e.g. measured values, times), where applicable. Increments specified above should be used. When only a small number of cases is presented, the respective values or time course can be presented individually.
(36) Data on SGA obtained from subjects receiving a vaccine should be compared with those obtained from an appropriately selected and documented control group(s) to assess background rates of hypersensitivity in non-exposed populations, and should be analysed by study arm and dose where possible, e.g. in prospective clinical trials.

### 4.1 Data presentation

These guidelines represent a desirable standard for the presentation and publication of data on SGA following immunisation to allow for comparability of data, and are recommended as an addition to data presented for the specific study question and setting. Additionally, it is recommended to refer to existing general guidelines for the presentation and publication of randomised controlled trials, systematic reviews, and meta-analyses of observational studies in epidemiology (e.g. statements of Consolidated Standards of Reporting Trials (CONSORT), of Improving the quality of reports of meta-analyses of randomised controlled trials (QUORUM), and of meta-analysis Of Observational Studies in Epidemiology (MOOSE), respectively) [94], [95], [96].

(37) All reported events of SGA should be presented according to the categories listed in guideline 31.
(38) Data on possible SGA events should be presented in accordance with data collection guidelines 1–24 and data analysis guidelines 31–36.
(39) Terms to describe SGA such as “low-grade”, “mild”, “moderate”, “high”, “severe” or “significant” are highly subjective, prone to wide interpretation, and should be avoided, unless clearly defined.
(40) Data should be presented with numerator and denominator (n/N) (and not only in percentages), if available.

Although immunisation safety surveillance systems denominator data are usually not readily available, attempts should be made to identify approximate denominators. The source of the denominator data should be reported and calculations of estimates be described (e.g. manufacturer data like total doses distributed, reporting through Ministry of Health, coverage/population based data, etc.).

(41) The incidence of cases in the study population should be presented and clearly identified as such in the text.
(42) If the distribution of data is skewed, median and range are usually the more appropriate statistical descriptors than a mean. However, the mean and standard deviation should also be provided.
(43) Any publication of data on SGA should include a detailed description of the methods used for data collection and analysis as possible. It is essential to specify:
  • The study design;
  • The method, frequency and duration of monitoring for SGA;
  • The trial profile, indicating participant flow during a study including drop-outs and withdrawals to indicate the size and nature of the respective groups under investigation;
  • The type of surveillance (e.g. passive or active surveillance);
  • The characteristics of the surveillance system (e.g. population served, mode of report solicitation);
  • The search strategy in surveillance databases;
  • Comparison group(s), if used for analysis;
  • The instrument of data collection (e.g. standardized questionnaire, diary card, report form);
  • Whether the day of immunisation was considered “day one” or “day zero” in the analysis;
  • Whether the date of onset^5^ and/or the date of first observation^6^ and/or the date of diagnosis^7^ was used for analysis; and
  • Use of this case definition for SGA, in the abstract or methods section of a publication.^15^

## Disclaimer

The findings, opinions and assertions contained in this consensus document are those of the individual scientific professional members of the working group. They do not necessarily represent the official positions of each participant’s Organization (e.g., government, university, or corporation). Specifically, the findings and conclusions in this paper are those of the authors and do not necessarily represent the views of their respective institutions.

## Footnotes

3 The case definition should be applied when there is no clear alternative diagnosis for the reported event to account for the combination of symptoms.

4 If Physical exam and ultrasound do not correlate, default to ultrasound.

5 If the reporting centre is different from the vaccinating centre, appropriate and timely communication of the adverse event should occur.

6 The date and/or time of onset is defined as the time post immunisation, when the first sign or symptom indicative for SGA occurred. This may only be possible to determine in retrospect.

7 The date and/or time of first observation of the first sign or symptom indicative for SGA can be used if date/time of onset is not known.

8 The date of diagnosis of an episode is the day post immunisation when the event met the case definition at any level.

9 The end of an episode is defined as the time the event no longer meets the case definition at the lowest level of the definition.

10 E.g. recovery to pre-immunisation health status, spontaneous resolution, therapeutic intervention, persistence of the event, sequelae, death.

11 An AEFI is defined as serious by international standards if it meets one or more of the following criteria: (1) it results in death, (2) is life-threatening, (3) it requires inpatient hospitalisation or results in prolongation of existing hospitalisation, (4) results in persistent or significant disability/incapacity, (5) is a congenital anomaly/birth defect, (6) is a medically important event or reaction.

12 To determine the appropriate category, the user should first establish, whether a reported event meets the criteria for the lowest applicable level of diagnostic certainty, e.g. Level three. If the lowest applicable level of diagnostic certainty of the definition is met, and there is evidence that the criteria of the next higher level of diagnostic certainty are met, the event should be classified in the next category. This approach should be continued until the highest level of diagnostic certainty for a given event could be determined. Major criteria can be used to satisfy the requirement of minor criteria. If the lowest level of the case definition is not met, it should be ruled out that any of the higher levels of diagnostic certainty are met and the event should be classified in additional categories four or five.

13 If the evidence available for an event is insufficient because information is missing, such an event should be categorised as “Reported SGA with insufficient evidence to meet the case definition”.

14 An event does not meet the case definition if investigation reveals a negative finding of a necessary criterion (necessary condition) for diagnosis. Such an event should be rejected and classified as “Not a case of SGA”.

15 Use of this document should preferably be referenced by referring to the respective link on the Brighton Collaboration website (http://www.brightoncollaboration.org).
